# GNSS-derived metrics as indicators of gastrointestinal parasitism in naturally infected grazing ewes and lambs

**DOI:** 10.3389/fvets.2026.1983272

**Published:** 2026-10-26

**Authors:** Diógenes Adriano Duarte Santana, Saulo Henrique Weber, Kassy Gomes da Silva, Carolina Schult Rieke, Julia Canha Consoni, Eduardo Mendonça Scheeren, Rüdiger Daniel Ollhoff, Cristina Santos Sotomaior

**Affiliations:** 1 Graduate Program in Animal Science, School of Medicine and Life Sciences, Pontifícia Universidade Católica do Paraná (PUCPR), Curitiba, Paraná, Brazil; 2 Department of Veterinary Medicine, School of Medicine and Life Sciences, Pontifícia Universidade Católica do Paraná (PUCPR), Curitiba, Paraná, Brazil; 3 Graduate Program in Health Technology, Polytechnic School, Pontifícia Universidade Católica do Paraná (PUCPR), Curitiba, Paraná, Brazil

**Keywords:** GNSS tracker, grazing sheep, *Haemonchus contortus*, precision livestock farming, TST

## Abstract

Gastrointestinal nematodes, particularly *Haemonchus contortus*, cause significant health and productivity losses in sheep. While Targeted Selective Treatment (TST) mitigates anthelmintic resistance by treating only individuals requiring clinical intervention, its large-scale implementation is limited by high labour demands. Precision Livestock Farming (PLF) offers continuous monitoring alternatives to overcome this barrier. Therefore, this study evaluated the use of Global Navigation Satellite System (GNSS) trackers to identify locomotion pattern alterations associated with gastrointestinal parasitism in grazing sheep, aiming to assess its potential as a screening tool for TST. Two experiments evaluated 15 lactating ewes and 15 weaned lambs under natural infection conditions. Custom-collared GNSS trackers recorded spatial parameters during grazing, while packed cell volume (PCV) and faecal egg counts (FEC) were evaluated weekly to classify individual health status. Animals were evaluated from a post-anthelmintic ‘healthy’ baseline until they became ‘unhealthy’ (PCV < 22%, or PCV < 27% + FEC > 2000). *H. contortus* was the prevalent nematode genus in both cohorts (ewes = 96%, lambs = 93.8%). Analysis of GNSS-derived metrics revealed that unhealthy lambs exhibited significantly higher peak speed, overall speed, peak acceleration, and total active time than their healthy counterparts (*p* < 0.05). In ewes, poor health status also significantly increased active time and overall speed (*p* < 0.05); however, peak speed and acceleration were influenced primarily by body weight rather than health status. GNSS metrics are promising non-invasive candidate indicators for TST in grazing sheep.

## 1 Introduction

Sanitary challenges, particularly gastrointestinal parasitism, are major limitations in sheep production, directly compromising animal health, welfare, and overall productivity. Gastrointestinal nematodes, such as *Haemonchus contortus*, cause significant clinical impacts, including anaemia, diarrhoea, and weight loss, which can lead to high mortality rates in lactating ewes and weaned lambs (1). To mitigate the selection for anthelmintic resistance and improve flock management, Targeted Selective Treatment (TST) has emerged as an important management strategy, identifying only those individuals requiring clinical intervention (2). Although TST can reduce unnecessary anthelmintic use and mitigate selection for anthelmintic resistance, its implementation at the flock level depends on efficiently identifying which animals require anthelmintic treatment. Conventional approaches often involve repeated handling and individual clinical evaluations, such as FAMACHA or body condition scoring, which increase labour requirements and potentially limit the frequency of assessments (3).

Precision Livestock Farming (PLF) technologies offer a non-invasive alternative for real-time continuous monitoring. By identifying changes in locomotion and behavioural patterns, automated monitoring could help direct attention towards individual sheep requiring further clinical or parasitological investigation, without replacing established assessment criteria. Recent studies have demonstrated that gastrointestinal nematode infections are associated with detectable alterations in sheep activity patterns, such as changes in lying behaviour and reduced overall movement (4, 5). While Global Navigation Satellite System (GNSS) technology has been successfully applied in other contexts, such as detecting parturition in sheep (6), its potential to identify movement signatures associated with gastrointestinal parasitism remains insufficiently explored. GPS-based studies have reported associations between parasitism and animal movement, including a positive relationship between faecal egg counts and distance travelled in a flock in which *Trichostrongylus* spp. predominated (7). Subsequently, GPS collars were integrated with parasitological data to investigate how grazing distribution varied according to health status and environmental conditions (8). Furthermore, recent research in lambs experimentally infected with *Teladorsagia circumcincta* demonstrated that gastrointestinal parasitism can produce behavioural alterations relevant to early identification, highlighting the need to further investigate remote monitoring of such indicators (9). In particular, *H. contortus* has received comparatively less attention in sensor-based research, with previous work relying on tri-axial accelerometers rather than GNSS-derived locomotion metrics (10). Thus, the potential of GNSS-derived locomotion metrics to characterize movement alterations associated with gastrointestinal parasitism, including situations involving *H. contortus*, remains poorly explored.

By enabling the detection of changes in health status, these technologies could reduce the need for manual handling, thereby lowering labour requirements for farmers while supporting more judicious anthelmintic use (11, 12). Therefore, the objective of this study was to evaluate the use of a GNSS tracker to identify alterations in locomotion patterns associated with gastrointestinal parasitism in sheep.

## 2 Method

### 2.1 Animals, management, and diet

The study was conducted at the sheep farming sector of the university farm of the Pontifícia Universidade Católica do Paraná (PUCPR), located in Fazenda Rio Grande, Brazil (25.66°S, 49.27°W). The research comprised two consecutive experimental phases using the same methodological framework: the first involving lactating ewes and the second involving weaned lambs.

#### 2.1.1 Ewe experimental phase

Fifteen lactating crossbred ewes (Ile de France or Texel x Hampshire Down), aged between 3 and 6 years, were evaluated. The ewes were at a similar physiological stage, entering the study (Day 0—D0) at a mean of 4.87 ± 1.25 days postpartum, with an initial body weight of 81.6 ± 13.7 kg. The experimental period lasted a total of 63 days. On D0 of the experiment, all ewes received a dose of broad-spectrum anthelmintic containing Nitroxynil (6.8 mg/kg) and Ivermectin (0.2 mg/kg).

The daily management routine was structured as follows: each morning, the ewes and their lambs were released from their collective pens and, to access the grazing area, they had to traverse a 160 m corridor. Upon arrival at the destination, the ewes remained restricted within 50 × 50 m paddocks for a daily grazing period of 6 h (Figure 1A). These paddocks consisted of annual ryegrass (*Lolium multiflorum* Lam.) and oats (*Avena sativa* L.) pastures, integrated with *Eucalyptus saligna* (4×4 m initial spacing, 3 years of age, covering a 1,000 m^2^ area of the paddock). In the late afternoon, the animals returned to the housing unit via the same corridor. The time spent traversing the corridor during these two specific transit moments was included in the total daily grazing time calculation. Upon returning to the collective pens at night, the ewes received corn silage supplemented with concentrate feed to meet nutritional requirements (13). Water was available *ad libitum* in both the paddocks and the collective pens.

**Figure 1:**
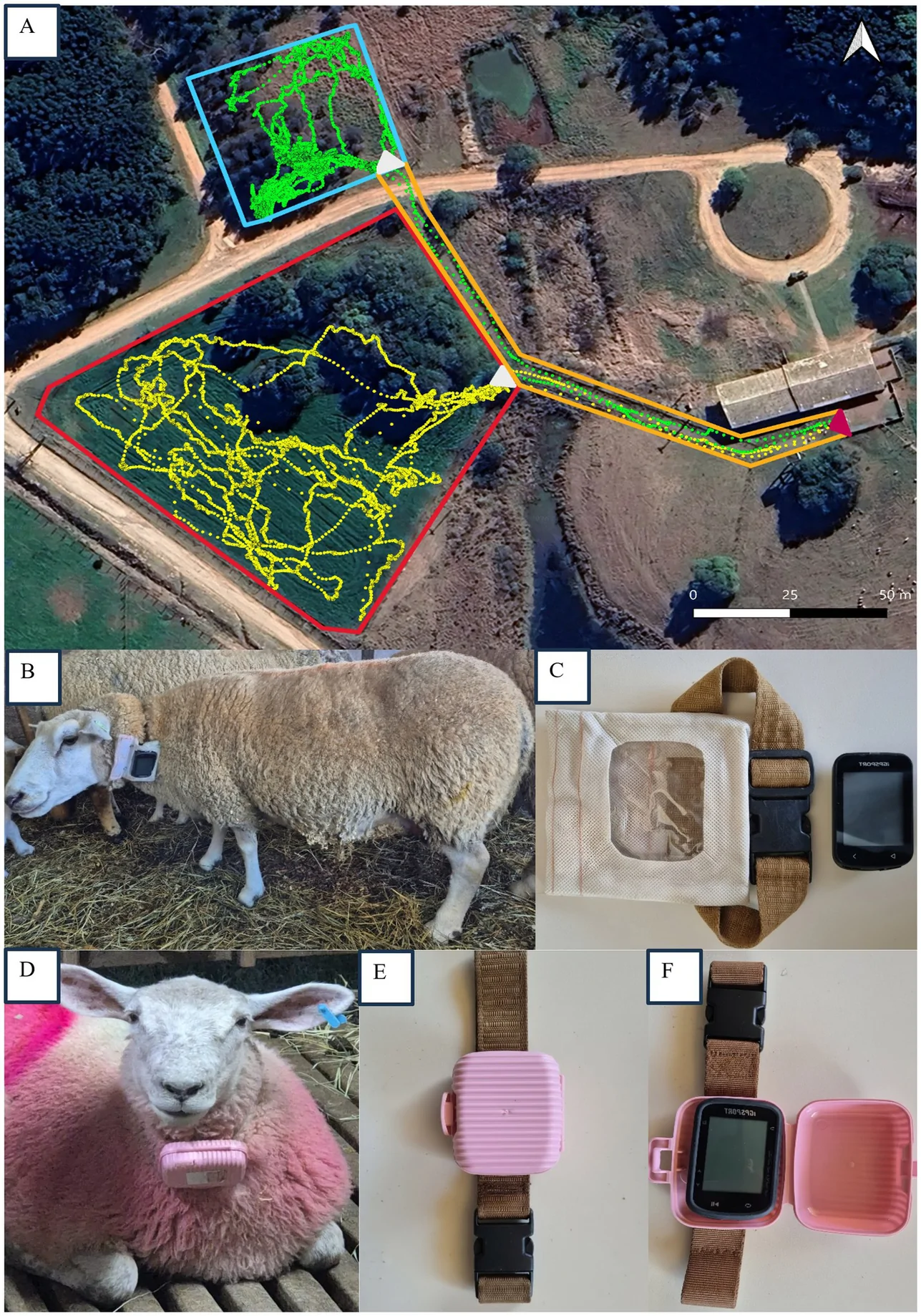
Layout of the experimental area and methods of attaching the Global Navigation Satellite System (GNSS) tracker for displacement monitoring. **(A)** Satellite view of the research facility (scale bar = 50 m), indicating the collective housing unit’s exit and entry point (fuchsia triangle), the transit corridor (orange outline) leading to the pastures, the paddock entrances (white triangles), the specific grazing area utilized by the lactating ewes (blue outline), and the distinct grazing paddock utilized by the weaned female lambs (red outline). Also shown are the GPS track points for the lactating ewes (green points) and for the weaned female lambs (yellow points). **(B)** Lactating ewe fitted with a custom neck collar containing the GNSS tracker housed within a non-woven fabric pouch. **(C)** Detailed view of the ewe’s collar assembly, displaying the adjustable webbing strap, quick-release buckle, non-woven fabric pouch with a transparent window, and the GNSS tracker. **(D)** Weaned female lamb fitted with a custom neck collar containing the GNSS tracker housed within an adapted rigid polypropylene container. **(E)** Detailed view of the lamb’s collar assembly with the rigid container closed. **(F)** Detailed view of the lamb’s collar assembly with the rigid container open, showing the internal placement of the GNSS tracker.

#### 2.1.2 Lamb experimental phase

Following the ewe trial, a second experiment was conducted using 15 crossbred weaned female lambs of the same genetic composition. The female lambs started the experiment at 82.4 ± 2.3 days of age (D0), with an initial body weight of 30.8 ± 4.8 kg. This second experimental period lasted a total of 91 days. 7 days prior to the trial (D-7), lambs received an anthelmintic treatment comprising Closantel (7.5 mg/kg) and Moxidectin (0.2 mg/kg).

The grazing area for lambs was an 8,544 m^2^ paddock of *Panicum maximum* cv. Atlas pasture, which included a 1,500 m^2^ area of native trees (Figure 1A). To access these grazing areas, lambs traversed the same physical corridor as the ewes but covered a shorter distance of 90 m, because their paddock was closer to the housing unit. Upon arrival, the lambs remained in the pasture for a daily grazing period of 4 h. At night, the weaned lambs were housed in collective pens and received a basal diet of corn silage supplemented with concentrate feed to meet nutritional requirements (13). Similar to the ewe phase, water was available *ad libitum* in both the paddocks and the collective pens.

In both experimental phases, the animals had daily access to pasture, naturally exposing them to gastrointestinal parasites, as the paddocks had been previously contaminated by other infected sheep.

### 2.2 Haematological and parasitological parameters

Laboratorial monitoring was performed weekly from D0 to the end of each experimental period (D63 for ewes and D91 for lambs). For haematological analysis, 4 mL of blood were collected from each sheep via jugular venipuncture into EDTA tubes for packed cell volume (PCV) determination using the micro-haematocrit method (centrifugation at 12,000 rpm for 5 min). Parasitological evaluations were also conducted every 7 days; individual faecal samples were collected directly from the rectum for faecal egg counts (FEC) using the modified Gordon and Whitlock (14) method, with a sensitivity of 50 eggs per gram (EPG). Third-stage larvae recovered from pooled group coprocultures (15) were morphometrically identified to the genus level (16).

### 2.3 Data acquisition and geospatial processing

Displacement was monitored using an iGPSPORT BSC200 (China) device. It was selected due to its 30-h continuous battery life, direct data extraction capability without reliance on third-party software, and multi-GNSS technology (GPS, GLONASS, Galileo, BeiDou, and QZSS) ensuring high-precision positioning. Preliminary equipment tests indicated a data loss rate of less than 1% at a sampling frequency of 1 Hz.

For the ewes, the devices were placed in 10 × 15 cm non-woven fabric (TNT) pouches. For the lambs, the devices were wrapped in adapted rigid polypropylene containers (9.5 × 7.5 × 3.5 cm). In both experimental phases, these enclosures were attached to custom-made collars 30 mm wide (100% polypropylene composition), with an individual adjustment system for each animal’s neck and positioned ventrally (Figures 1B–F).

Data acquisition was strictly limited to the period when sheep were in the pasture. Recording began and ended exactly when the animals crossed the corridor gate leading to the paddocks, ensuring no data was collected while the animals were in the housing unit. To ensure systematic data collection, sensors were deployed 4 days a week. Consequently, monitoring totalled 36 days for the ewes and 52 days for the lambs within their respective experimental timeframes.

Geospatial processing and the extraction of positional error parameters (Q1, Q3, IQR) were performed using QGIS software (version 4.2.0). From the raw GNSS data, spatial coordinates were obtained and projected to the SIRGAS 2000/UTM zone 22S (EPSG:31982) system. These projected coordinates (X, Y, and Z axes) and timestamps were used to mathematically derive distance, velocity, and acceleration parameters for each animal.

To accurately quantify actual movement and mitigate artificial signal noise (drift) inherent to GNSS trackers when stationary, a static displacement threshold was established. Using the Tukey method for outlier detection, based on the interquartile range of positional errors derived from stationary device tests, a threshold of 0.20 m was calculated. Consequently, since coordinates were recorded every second, consecutive displacements corresponding to a speed lower than 0.20 m/s was reclassified as zero (stationary behaviour), ensuring that only genuine locomotion was computed in the final analysis (17).

The GNSS-derived metrics that were analysed were: Inactive Time (%), the sum of time intervals during which the distance travelled per second was zero; Active Time (%), the sum of time intervals during which the distance travelled per second was greater than zero; Total Distance (km), the sum of distances travelled across all recorded time intervals; Moving Speed (km/h), the distance travelled during each time interval divided by the corresponding time interval; Peak Speed (km/h), the maximum speed recorded during the evaluation day; Moving Acceleration (m/s^2^), the rate of change of movement speed between consecutive observations; and Peak Acceleration (m/s^2^), the maximum acceleration recorded during the evaluation day.

Active Time and Inactive Time represent strictly GNSS-derived locomotor displacement based on the established velocity thresholds, rather than the direct identification of specific ethological behaviours such as grazing, resting, or rumination.

The inclusion of Peak Speed and Peak Acceleration was established as a study-specific analytical hypothesis. While previous research has demonstrated an association between FEC and mean distance travelled in sheep, the high variability observed around these models suggests that basic mean movement metrics alone might not fully capture the complexity of locomotion (7). Drawing on locomotor studies across different species, maximal velocity metrics are frequently utilized to better characterize individual movement differences that mean values fail to explain (18). Furthermore, peak speed and maximum acceleration have been identified as highly discriminative features for classifying active behaviours such as grazing and walking in pasture-based ruminants (19). Based on this rationale, we hypothesized that transient, high-intensity locomotor responses (peaks) could offer additional sensitivity to the physiological constraints imposed by gastrointestinal parasitism. Mean values, in contrast, may attenuate short-term variations by averaging periods of both high and low activity, potentially reducing their ability to reflect associations with FEC and PCV.

Because pasture exposure time varied among observation days, total distance travelled was not used as a primary movement metric. Instead, movement was normalized to observation duration and expressed as overall speed. Likewise, activity was expressed as the percentage of observation time spent active or inactive rather than as absolute duration.

### 2.4 Definition of healthy and unhealthy sheep

To evaluate the effect of parasitic infection on intra-individual sensor GNSS-derived metrics, two distinct time points were established during the experimental period. The ‘healthy’ baseline was defined as D7 for ewes (one-week post-anthelmintic treatment) and D0 for lambs (treated 7 days prior to the trial). Conversely, the ‘unhealthy’ time point was defined as the moment an animal presented a PCV < 22%, or a PCV < 27% combined with a FEC > 2000 EPG. These clinical thresholds were based on anaemia indicators anchored in the limits of the FAMACHA system (20), where scores F1 (PCV ≥ 28%) and F2 (PCV ≥ 23%) are considered healthy. Animals that did not reach these parasitological thresholds were classified as healthy throughout the study. The daily GNSS-derived metrics extracted for statistical analysis were those recorded on the day immediately following each defined ‘healthy’ or ‘unhealthy’ time point.

### 2.5 Statistical analysis

Statistical analyses of haematological (PCV) and parasitological (FEC) data, as well as correlations with sensor data, were performed using IBM SPSS statistics (version 25). FEC data were log-transformed [log_10_(x + 1)] to normalize the distribution and stabilize variances. Descriptive statistical analysis is presented in Supplementary Tables 1–3.

The relationship between FEC and PCV was assessed using Pearson’s correlation coefficient. FEC values were transformed using log₁₀(EPG + 10), and a linear regression model was fitted by the ordinary least squares method: PCV = a + b × log₁₀(EPG + 10). Model goodness of fit was assessed using the coefficient of determination (*R*^2^).

GNSS-derived metrics were compared between healthy and unhealthy groups using a Linear Mixed-Effects Model (LMM) to appropriately account for the repeated-measures structure of the unbalanced longitudinal data. All 15 animals from each phase were classified as healthy at baseline. During the experimental period, nine animals reached the predefined unhealthy threshold. The model was defined as Y = μ + G + βP + subject + ε, where Y represents the specific GNSS-derived metric, μ is the overall intercept, G is the fixed effect of the health group (healthy or unhealthy), P is the fixed covariate of body weight (kg) with β as its regression coefficient, subject is the random effect of the individual animal to account for the repeated observations from animals that transitioned from the healthy to the unhealthy state, and ε is the residual error.

To assess the influence of individual GNSS-derived metrics on PCV and FEC, two separate general linear models (GLMs) were fitted, one using PCV and the other using FEC as the dependent variable. The models were specified as Y = μ + aPS + bPA + cOS + dIT + ε, where *Y* represents PCV or FEC, PS is peak speed, PA is peak acceleration, OS is overall speed, IT is inactive time, μ is the overall mean, *a–d* are the regression coefficients associated with each GNSS-tracker parameter, and ε is the residual error.

## 3 Results

The mean PCV values of ewes and lambs varied from 25.4 to 34.8% (Supplementary Table 1), with minimum and maximum individual values between 15 and 44%. For the FEC, mean values were between 183.3 and 5226.7 EPG, varying from zero to 38,400 EPG. The genus *Haemonchus* (ewes = 96%, lambs = 93.8%) was the most prevalent in coproculture, followed by the genus *Trichostrongylus* (ewes = 4%, lambs = 6.2%). A negative log-linear relationship was observed between PCV and log-transformed FEC [log (EPG + 10)] across both age groups. The rate of PCV decline in response to increasing parasite load was parallel, demonstrating an equivalent slope for both lambs and ewes (Figure 2). Throughout the experimental period, the lambs exhibited an average daily weight gain of 124 ± 30 g/d, reaching a final body weight of 42.1 ± 5.4 kg.

**Figure 2:**
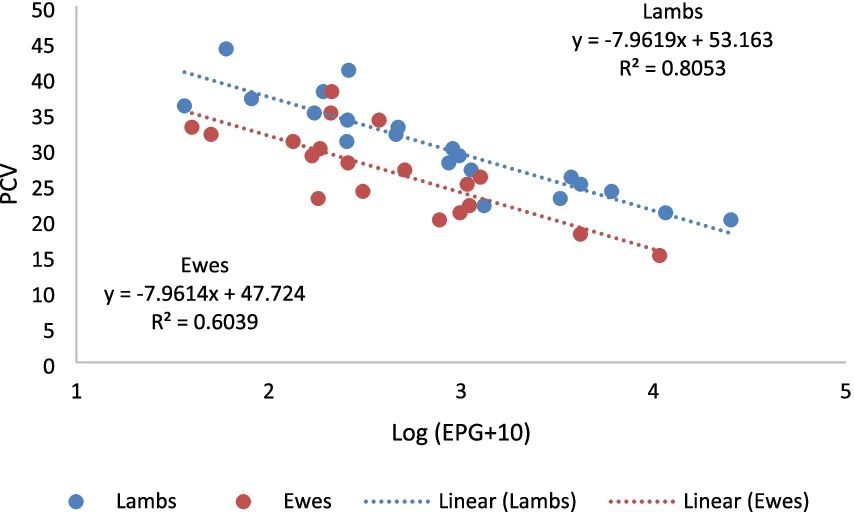
Linear regression analysis of packed cell volume (PCV, in %) versus log-transformed fecal egg counts [Log (EPG + 10)] in ewes and lambs.

The mean time ewes and lambs were evaluated by GNSS trackers was 410.4 ± 37.8 min (6.8 ± 0.6 h) and 255.4 ± 53.8 min (4.3 ± 0.9 h) per day, respectively. The descriptive statistics of all the parameters can be seen in Supplementary Tables 2, 3. When the GNSS-derived parameters were used in GLM, the observed values of PCV and FEC and model-predicted estimates demonstrated a consistent positive fit across both biological parameters (Figure 3).

**Figure 3:**
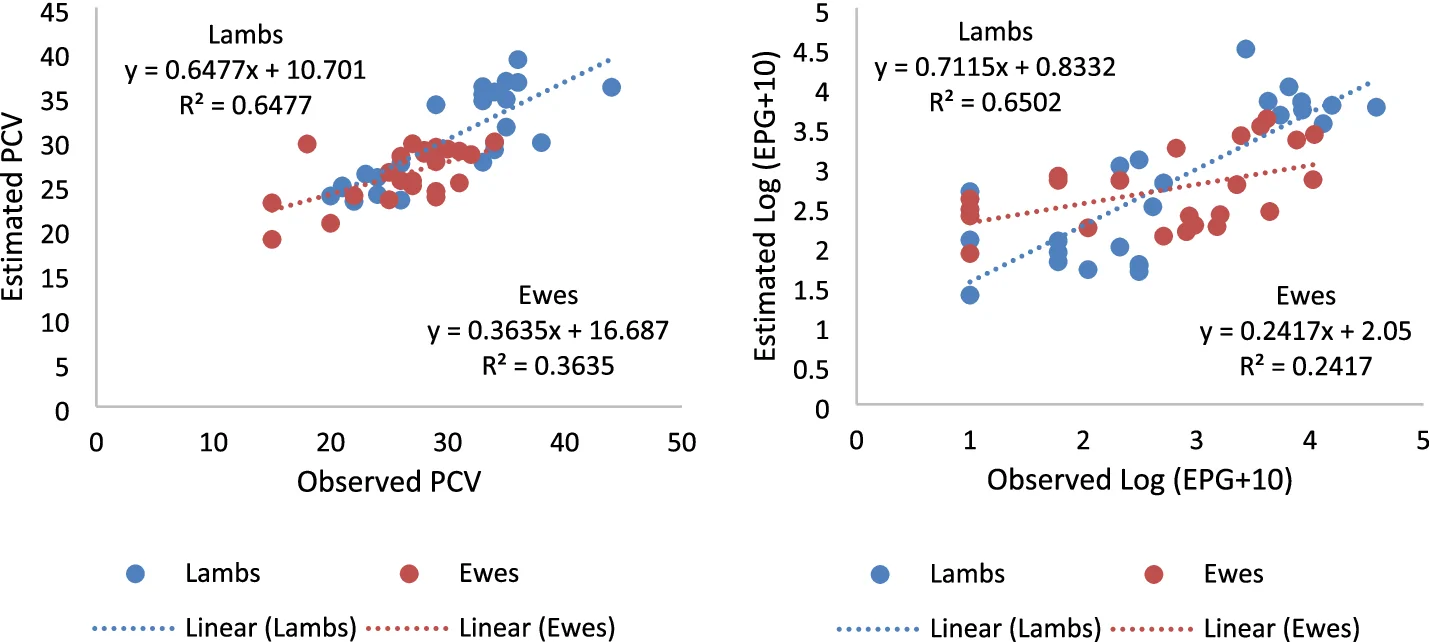
Linear regression analyses comparing observed and model-estimated values for packed cell volume [PCV, left panel] and log-transformed fecal egg counts [Log (EPG + 10), right panel] derived from GNSS-derived metrics in lactating ewes and weaned lambs.

Based on the established threshold for classifying sheep as unhealthy, nine lambs and nine ewes met this criterion during the experimental phase. When we compare the mean values of healthy and unhealthy animals, the clinical and parasitological parameters differed significantly in both age cohorts (*p* < 0.001). Unhealthy lambs exhibited an increase in FEC (11,433.33 ± 10,912.72 EPG vs. 183.33 ± 161.10 EPG) and a corresponding reduction in PCV (23.00 ± 2.18% vs. 34.80 ± 3.23%). Similarly, unhealthy ewes presented significantly elevated FEC (5,277.78 ± 3,561.17 EPG vs. 410.71 ± 495.43 EPG) and diminished PCV (21.56 ± 4.77% vs. 29.21 ± 2.29%) compared to healthy ewes.

In relation to the GNSS-derived parameters, the health status was significantly associated with differences in GNSS-derived movement metrics, mainly in lambs (Table 1). Unhealthy lambs exhibited significantly higher overall speed, peak speed, peak acceleration, and total active time compared to their healthy counterparts. In ewes, health status also significantly affected overall speed and activity duration, as unhealthy ewes demonstrated increased active time. However, unlike the younger cohort, peak speed and acceleration in ewes were not significantly influenced by health status but were instead significantly affected by body weight.

**Table 1:** Mean (± standard deviation) of GNSS-derived parameters values from lambs and ewes, according to healthy and unhealthy groups.

GNSS-derived parameters	Lambs				Ewes			
	Healthy (*n* = 15)	Unhealthy (*n* = 9)	*p*-value: group	*p*-value: weight	Healthy (*n* = 15)	Unhealthy (*n* = 9)	*p*-value: group	*p*-value: weight
Peak speed (km/h)	17.58 ± 1.84	25.44 ± 7.66	0.019	0.553	17.13 ± 2.76	18.26 ± 4.62	0.405	0.006
Peak acceleration (m/s^2^)	1.88 ± 0.44	2.69 ± 1.20	0.022	0.343	1.62 ± 0.74	2.14 ± 1.10	0.137	0.011
Overall speed (km/h)	0.19 ± 0.06	0.42 ± 0.05	<0.001	0.931	0.17 ± 0.03	0.22 ± 0.06	0.006	0.999
Active time (%)	8.55 ± 2.34	17.02 ± 2.03	<0.001	0.931	8.14 ± 1.82	11.41 ± 4.70	0.031	0.644

## 4 Discussion

Wearable sensor technologies have the capability of automatically detecting behavioural changes of animals in real-time, which may enable early and timely detection of parasite infection, leading to improvements in treatment protocols (11, 21). While recent studies examining the association between sensors and gastrointestinal parasitism primarily employ accelerometers (5, 10, 22, 23), the application of GNSS sensors remains underexplored.

In the present study, GNSS-derived metrics were associated with health status under natural grazing conditions. Unhealthy ewes, characterized by higher FEC and lower PCV, exhibited higher overall speed and active time, while lambs also displayed higher peak speed and peak acceleration. This pattern contrasts with studies in which gastrointestinal parasitism was associated with reduced activity or increased resting behaviour (4, 10, 23) but is consistent with the positive association between FEC and distance travelled reported by Falzon et al. (7) using GPS-based monitoring. This apparent discrepancy may be related to the different behavioural dimensions captured by each technology: accelerometers quantify body movement and allow the distinction of behavioural states, whereas GNSS primarily measures the horizontal displacement of the animal within the grazing environment (24).

*H. contortus*, the prevalent hematophagous parasite in the evaluated flock, causes loss of blood, proteins, and nutrients, resulting in anaemia and metabolic stress (10, 25). Given this pathological condition, this increased time spent in motion could be explained as a hyperactive behaviour stemming from systemic discomfort and an inability to maintain continuous rest, rather than reflecting a healthier state or greater physiological vigour. While our GNSS data cannot directly assess resting-bout structure, this restlessness is corroborated by Williams et al. (5), who observed a higher frequency of short lying bouts in ewes with higher FECs. We propose that under grazing conditions, this restlessness could contribute to the greater active time and increased speed recorded by GNSS tracking in the present study. This proposed pattern aligns with the positive association between FEC and distance travelled documented by Falzon et al. (7), as well as the increased time spent in active and highly active behaviours by infected lambs reported by Fan et al. (22).

Because GNSS tracking primarily records spatial displacement rather than direct ethological states, the specific behavioural mechanisms driving the increased peak speed and acceleration are formulated as explanatory hypotheses. The peak speed may relate to the search for grazing sites with lower parasitic risk, as sheep modify spatial utilization to avoid faeces-contaminated patches (26). Additionally, we also hypothesize that acceleration peaks could reflect frequent behavioural shifts, such as changes in direction or avoidance of conspecifics, rather than continuous displacement. This aligns with Fogarty et al. (10), who identified movement variation and peak acceleration as key variables for classifying infected sheep. An additional explanation may involve alterations in the temporal organization of locomotion. The combined physiological burden associated with reduced PCV and elevated FEC may shift movement behaviour toward shorter displacement bouts, thereby increasing the occurrence of higher instantaneous speeds and accelerations.

Although our study did not experimentally compare different levels of infection severity, the direction and magnitude of locomotor variations likely depend on this factor. In our flock, the lowest PCV recorded was 15% for ewes and 20% for lambs, indicating that the animals did not reach a state of extreme anaemia and profound weakness. We hypothesize that during this initial stage of clinical impairment, the physiological demands of parasitism may elicit acute compensatory locomotor adaptations—such as the elevations in speed and active time observed in our study. Conversely, in cases of greater severity, animals are expected to adopt energy conservation strategies, ultimately resulting in an absence of movement or significantly reduced overall activity. In support of this literature-derived hypothesis, Fogarty et al. (10) reported a negative correlation between FEC and activity, and a positive correlation between PCV and activity. Furthermore, Ikurior et al. (4) observed altered movement profiles in infected lambs, while Hempstead et al. (27) demonstrated that *H. contortus* infection induces dose-dependent reductions in erythrocytes, haemoglobin, and haematocrit, driving severe physiological adaptations prior to the manifestation of profound lethargy.

The observed GNSS-derived locomotor patterns associated with parasitism differed between age categories. Lambs exhibited elevations in speed and acceleration, whereas adult ewes maintained stable peak metrics. Because ewes and lambs were evaluated in consecutive experiments under distinct environmental and management conditions (e.g., pasture type, paddock characteristics, and grazing duration), these variations cannot be attributed exclusively to age. Nevertheless, one potential explanation is immunological immaturity. Since lambs are still developing acquired immunity, they present greater susceptibility to gastrointestinal nematodes. The immunological immaturity and the severity of anaemia in young animals necessitate acute compensatory locomotor adaptations, reflecting individual variation in disease response (5, 10, 25). While age and physiological category can influence baseline haematological parameters and FEC dynamics, we applied uniform clinical thresholds to classify both cohorts as ‘unhealthy’. The use of common thresholds represents an operational definition adopted to standardize the intervention criteria for both highly susceptible categories (lactating ewes and weaned lambs), rather than implying an exactly equivalent biological severity.

While our results demonstrate significant associations between GNSS-derived metrics and health status, the potential confounding effect of experimental time must be acknowledged. The ‘healthy’ baseline was established at the beginning of the trial, whereas the ‘unhealthy’ measurements occurred subsequently as the natural infection progressed (within a maximum window of 28 days among animals). Although body weight was included as a covariate in our statistical models to account for growth-related physical changes, particularly relevant for growing lambs, the observed locomotor variations could partially reflect other unmeasured time-related factors, such as behavioural adaptation to the paddock or minor fluctuations in environmental and pasture conditions. Future longitudinal studies incorporating parallel uninfected control groups would be beneficial to completely isolate the locomotor impacts of gastrointestinal parasitism from temporal environmental changes.

Although these are preliminary results with a limited number of ewes and lambs evaluated, the detection of deviations in GNSS-derived movement metrics highlights these parameters as potential candidate indicators for future TST screening systems. If validated by future diagnostic performance evaluations, such systems could offer potential benefits, including reduced reliance on manual clinical assessments and the mitigation of anthelmintic resistance. Ultimately, this brief research report demonstrates the potential of on-animal GNSS-derived movement metrics to detect changes in locomotion caused by nematode parasitism in grazing sheep.

## Data Availability

The raw data supporting the conclusions of this article will be made available by the authors, without undue reservation.
