# Supplementary material for: GNSS-derived metrics as indicators of gastrointestinal parasitism in naturally infected grazing ewes and lambs
Source: Front Vet Sci. 2026 Oct 26;13:1983272. doi: 10.3389/fvets.2026.1983272 (PMC13745253; doi:10.3389/fvets.2026.1983272)
Supplement: Supplementary file 1 [file Table_1.docx]

Supplementary Table 1 - Means of fecal egg counts (FEC, in eggs per gram - EPG) and packed cell volume (PCV, in %) levels of ewes and lambs

| Days | Ewes | | Lambs | |
| --- | --- | --- | --- | --- |
|  | FEC | PCV (%) | FEC | PCV (%) |
| D0 | 1904.2 ± 1883.4 | 29.3 ± 4.8 | 183.3 ± 161.1 | 34.8 ± 3.2 |
| D7 | 386.7 ± 486.4 | 28.8 ± 2.7 | 490.0 ± 557.5 | 32.5 ± 2.4 |
| D14 | 250.0 ± 358.1 | 30.3 ± 3.8 | 720.0 ± 680.3 | 34.3 ± 2.5 |
| D21 | 520.0 ± 738.4 | 27.0 ± 3.9 | 716.7 ± 571.9 | 32.0 ± 2.3 |
| D28 | 2293.3 ± 3574.9 | 25.5 ± 5.7 | 696.7 ± 501.2 | 32.7 ± 2.9 |
| D35 | 1226.7 ± 1514.8 | 25.4 ± 3.3 | 1150.0 ± 1048.0 | 30.7 ± 2.7 |
| D42 | 1160.0 ± 1313.8 | 25.8 ± 2.7 | 1966.7 ± 1865.1 | 28.7 ± 2.8 |
| D49 | 1603.3 ± 2091.3 | 25.7 ± 4.0 | 3290.0 ± 4269.0 | 27.3 ± 3.5 |
| D56 | 1900.0 ± 3329.2 | 26.9 ± 4.2 | 5226.7 ± 9463.9 | 27.9 ± 3.5 |
| D63 | 510.0 ± 463.0 | 28.3 ± 3.3 | 3663.3 ± 4631.7 | 27.8 ± 2.4 |
| D70 |  |  | 2406.7 ± 3515.9 | 28.3 ± 3.5 |
| D77 |  |  | 3683.3 ± 5021.3 | 27.7 ± 4.1 |
| D84 |  |  | 2813.3 ± 2962.2 | 26.9 ± 3.8 |
| D91 |  |  | 2286.7 ± 1727.1 | 29.3 ± 2.6 |
|  | | | | |

| Supplementary Table 2 - Weekly means of spatial and behavioural metrics in grazing ewes derived from GNSS tracking data. | | | | | | | |
| --- | --- | --- | --- | --- | --- | --- | --- |
| Week | IT  (min) | AT  (min) | TD  (km) | MS  (km/h) | OS  (km/h) | MA  (m/s^2^) | OA  (m/s^2^) |
| Week 1 | 366.9 ± 40.8 | 35.7 ± 11.9 | 1.129 ± 0.284 | 1.9 ± 1.9 | 0.2 ± 0.8 | 0.06 ± 0.20 | 0.00005 ± 0.07021 |
| Week 2 | 381.6 ± 34.1 | 35.3 ± 7.4 | 1.124 ± 0.173 | 1.9 ± 1.9 | 0.2 ± 0.8 | 0.06 ± 0.19 | 0.00006 ± 0.06829 |
| Week 3 | 407.6 ± 11.5 | 31.3 ± 8.0 | 1.062 ± 0.213 | 2.1 ± 2.0 | 0.1 ± 0.7 | 0.06 ± 0.20 | 0.00005 ± 0.06469 |
| Week 4 | 367.5 ± 35.3 | 32.7 ± 12.2 | 1.060 ± 0.272 | 2.0 ± 1.9 | 0.2 ± 0.7 | 0.06 ± 0.19 | 0.00007 ± 0.06677 |
| Week 5 | 362.1 ± 30.6 | 37.8 ± 14.6 | 1.243 ± 0.361 | 2.0 ± 1.9 | 0.2 ± 0.8 | 0.06 ± 0.20 | 0.00009 ± 0.07348 |
| Week 6 | 400.9 ± 56.8 | 34.8 ± 9.2 | 1.191 ± 0.260 | 2.1 ± 2.0 | 0.2 ± 0.8 | 0.06 ± 0.20 | 0.00006 ± 0.06778 |
| Week 7 | 354.4 ± 19.6 | 40.2 ± 11.2 | 1.358 ± 0.344 | 2.1 ± 1.9 | 0.2 ± 0.8 | 0.05 ± 0.20 | 0.00008 ± 0.07448 |
| Week 8 | 369.6 ± 37.6 | 35.4 ± 12.6 | 1.221 ± 0.314 | 2.1 ± 2.1 | 0.2 ± 0.8 | 0.05 ± 0.20 | 0.00006 ± 0.07114 |
| Week 9 | 364.7 ± 26.2 | 33.3 ± 6.9 | 1.180 ± 0.197 | 2.2 ± 2.0 | 0.2 ± 0.8 | 0.06 ± 0.21 | 0.00008 ± 0.07121 |
| IT = Inactive Time. AT = Active Time. TD = Total Distance. MS = Moving Speed. OS = Overall Speed. MA = Moving Acceleration. OA = Overall Acceleration | | | | | | | |

| Supplementary Table 3 - Weekly means of spatial and behavioural metrics in grazing lambs derived from GNSS tracking data. | | | | | | | |
| --- | --- | --- | --- | --- | --- | --- | --- |
| Week | IT  (min) | AT  (min) | TD  (km) | MS  (km/h) | OS  (km/h) | MA  (m/s^2^) | OA  (m/s^2^) |
| Week 1 | 218.8 ± 8.6 | 18.4 ± 4.3 | 0.665 ± 0.144 | 2.2 ± 2.3 | 0.2 ± 0.9 | 0.06 ± 0.24 | 0.0002 ± 0.0753 |
| Week 2 | 218.1 ± 6.9 | 24.3 ± 4.9 | 0.915 ± 0.183 | 2.3 ± 2.5 | 0.2 ± 1.0 | 0.05 ± 0.26 | 0.0002 ± 0.0919 |
| Week 3 | 219.8 ± 9.4 | 28.4 ± 7.8 | 0.959 ± 0.186 | 2.1 ± 2.0 | 0.2 ± 0.9 | 0.05 ± 0.23 | 0.0002 ± 0.0889 |
| Week 4 | 221.3 ± 7.0 | 25.6 ± 4.6 | 0.923 ± 0.124 | 2.2 ± 2.6 | 0.2 ± 1.0 | 0.06 ± 0.25 | 0.0002 ± 0.0927 |
| Week 5 | 220.0 ± 12.4 | 27.7 ± 5.0 | 1.036 ± 0.199 | 2.3 ± 2.4 | 0.3 ± 1.1 | 0.05 ± 0.25 | 0.0002 ± 0.0946 |
| Week 6 | 220.8 ± 11.5 | 28.0 ± 5.0 | 1.087 ± 0.160 | 2.3 ± 2.5 | 0.3 ± 1.1 | 0.05 ± 0.26 | 0.0002 ± 0.0959 |
| Week 7 | 211.6 ± 23.1 | 37.5 ± 7.9 | 1.475 ± 0.278 | 2.4 ± 2.4 | 0.4 ± 1.3 | 0.05 ± 0.26 | 0.0002 ± 0.1104 |
| Week 8 | 254.7 ± 58.0 | 52.8 ± 12.0 | 1.987 ± 0.475 | 2.3 ± 2.4 | 0.4 ± 1.3 | 0.05 ± 0.25 | 0.0002 ± 0.1150 |
| Week 9 | 195.4 ± 26.8 | 49.5 ± 14.7 | 1.763 ± 0.546 | 2.1 ± 2.5 | 0.4 ± 1.4 | 0.06 ± 0.25 | 0.0003 ± 0.1250 |
| Week 10 | 191.7 ± 16.9 | 44.9 ± 8.4 | 1.769 ± 0.251 | 2.4 ± 2.9 | 0.5 ± 1.6 | 0.06 ± 0.29 | 0.0003 ± 0.1376 |
| Week 11 | 232.0 ± 28.7 | 56.6 ± 11.8 | 2.140 ± 0.459 | 2.3 ± 2.3 | 0.4 ± 1.4 | 0.05 ± 0.25 | 0.0003 ± 0.1255 |
| Week 12 | 260.5 ± 43.5 | 50.7 ± 8.8 | 1.910 ± 0.292 | 2.3 ± 2.1 | 0.4 ± 1.2 | 0.05 ± 0.24 | 0.0002 ± 0.1108 |
| Week 13 | 225.1 ± 41.1 | 48.2 ± 10.1 | 1.921 ± 0.383 | 2.4 ± 2.5 | 0.4 ± 1.4 | 0.05 ± 0.28 | 0.0003 ± 0.1276 |
| IT = Inactive Time. AT = Active Time. TD = Total Distance. MS = Moving Speed. OS = Overall Speed. MA = Moving Acceleration. OA = Overall Acceleration | | | | | | | |
